# Ligamentum Arteriosum Calcification Associated With Kommerell Diverticulum Mimicking a Foreign Body in the Esophagus: A Case Report

**DOI:** 10.7759/cureus.85365

**Published:** 2025-06-04

**Authors:** Nobuhiro Takahashi, Akihiro Shimotakahara, Hirofumi Tomita

**Affiliations:** 1 Department of Surgery, Tokyo Metropolitan Children's Medical Center, Fuchu, Tokyo, JPN; 2 Department of Surgery, Tokyo Metropolitan Children’s Medical Center, Fuchu, Tokyo, JPN

**Keywords:** beef bone, esophageal foreign bodies, esophageal perforation, kommerell diverticulum, ligamentum arteriosum calcification

## Abstract

Esophageal perforations caused by a foreign body are a serious condition requiring urgent intervention due to the risk of severe mediastinitis. However, certain calcified anatomical structures may be misdiagnosed as a foreign body owing to the similarities of the features on imaging studies. We herein report the case of a seven-year-old female patient who presented with persistent pharyngeal discomfort after ingesting skewered beef containing bone fragments. Initial computed tomography (CT) at the previous hospital revealed a high-density mediastinal shadow, which raised suspicion of an esophageal perforation. Further evaluation at the study center identified a right aortic arch with a Kommerell diverticulum and a linear, high-density lesion between the diverticulum and the pulmonary artery in the area corresponding to the ductus arteriosus. In the absence of inflammatory signs, the lesion was diagnosed as ligamentum arteriosum calcification associated with a vascular ring. The patient was managed conservatively and discharged without complications. This case highlights the importance of recognizing that ligamentum arteriosum calcification can mimic esophageal perforation. Assessing whether the symptoms correlate with mediastinitis due to an esophageal perforation, along with the accurate interpretation of CT findings, including the anatomical location of the lesion and the absence of inflammatory signs, was crucial to avoid unnecessary invasive procedures.

## Introduction

Esophageal foreign bodies in children commonly occur in the hypopharynx or the upper thoracic esophagus. Perforation caused by a foreign body occurs in approximately 1-4% of cases and may lead to mediastinitis, a life-threatening condition characterized by chest pain, fever, and systemic inflammatory response. Management typically requires prompt surgical intervention and carries a high mortality rate [1,2]. Fish bones are one of the most frequent esophageal foreign bodies and typically appear as linear, high-density structures on computed tomography (CT) [2].

Vascular rings are rare, congenital aortic arch anomalies that occur at a frequency of <0.1% and can lead to compression of the trachea, esophagus, or both [3]. There are several types; the form characterized by a right aortic arch with a left ligamentum arteriosum is sometimes accompanied by a Kommerell diverticulum, an embryological remnant of the distal fourth aortic arch [3]. In the fetal circulatory system, the ductus arteriosus connects the pulmonary artery to the aorta, forming the ligamentum arteriosum after birth. Calcification of the ligamentum arteriosum, which can present a curvilinear, clumped, punctate, or linear appearance, occurs in 13.2-60.9% of the pediatric population and can occasionally be mistaken for esophageal foreign bodies such as fish bones on CT imaging, necessitating careful differentiation [4-7].

We herein report a pediatric case presenting with anterior chest pain following the ingestion of beef bone, in which esophageal perforation was initially suspected. CT demonstrated a linear calcification in the mediastinum, mimicking a foreign body. Further evaluation led to the diagnosis of ligamentum arteriosum calcification associated with a Kommerell diverticulum, thereby preventing unnecessary invasive intervention. Given that mediastinitis secondary to esophageal perforation can follow a fulminant course and demands urgent management, accurate radiological differentiation is of critical importance.

## Case presentation

**Figure 1:**
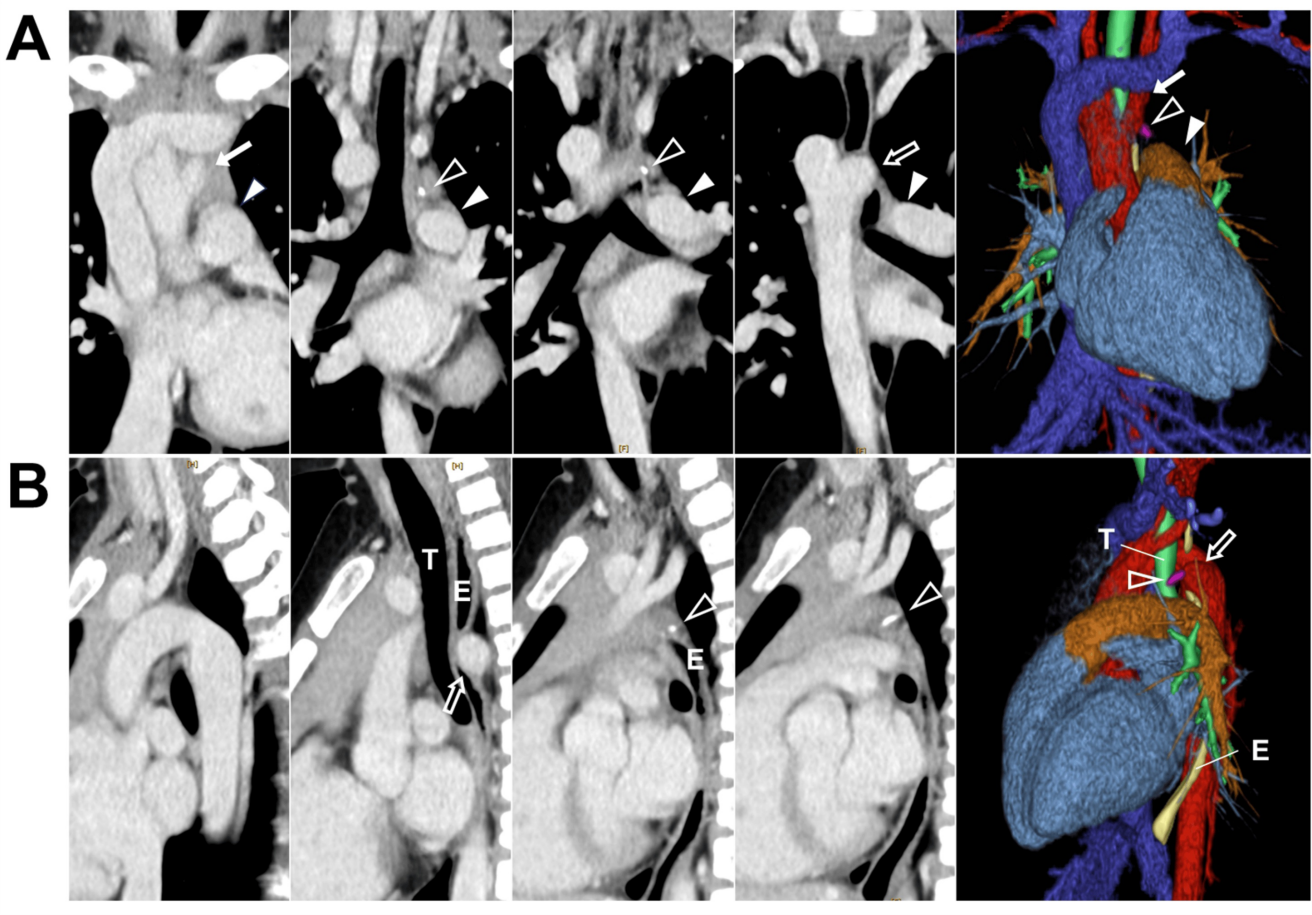
Contrast-enhanced cervicothoracic CT findings of ligamentum arteriosum calcification A. Coronal imaging. A linear, high-density lesion was observed between the Kommerell diverticulum and the pulmonary artery. Arrows indicate the left subclavian artery, arrowheads indicate the pulmonary artery, open arrow indicates the Kommerell diverticulum, and open arrowheads indicate calcification of the ligamentum arteriosum. B. Sagittal imaging. The Kommerell diverticulum can be seen compressing the trachea and esophagus from behind. Open arrows indicate Kommerell diverticulum, and open arrowheads indicate calcification of the ligamentum arteriosum. T, trachea; E, esophagus

A seven-year-old female patient presented with persistent pharyngeal discomfort following the ingestion of skewered beef, which was reported to contain bone fragments, according to her family. Upon noticing the presence of bones while eating, she partially expelled the ingested material. Subsequently, she developed pharyngeal discomfort, which gradually progressed to anterior chest discomfort over the course of two days, prompting evaluation at a referring hospital. No foreign body was identified in the pharynx or larynx; however, cervicothoracic CT revealed a high-density shadow in the mediastinum, raising suspicion of esophageal perforation secondary to a bone fragment. The patient was subsequently transferred to our institution, where she reported mild anterior chest pain, subjectively localized to the region of the suprasternal notch. The pain had been gradually improving by the time of presentation. Her vital signs were unremarkable, she was afebrile, and the inflammatory markers (white blood cell count of 8,180/μL and C-reactive protein level of 0.02 mg/dL) were normal. Considering these findings, although she reported anterior chest pain, the overall clinical presentation suggested only mild symptoms, making significant mediastinitis due to esophageal perforation less likely. Cervicothoracic CT revealed a right aortic arch without an aberrant left subclavian artery accompanied by a Kommerell diverticulum, and a linear, high-density lesion was observed between the diverticulum and the pulmonary artery (Figure 1). Since this corresponded to the location of the ductus arteriosus and there were no signs of inflammation in the surrounding area, the lesion was considered to be a calcification of the left ligamentum arteriosum associated with the vascular ring. The patient was admitted for observation. Her symptoms improved over the next day. While the cause of her chest pain remained unclear, esophageal perforation was denied, and she was discharged after resuming oral intake. In the absence of dysphagia, the patient is being managed via outpatient follow-up.

## Discussion

Perforations caused by an esophageal foreign body are associated with a high mortality rate and often necessitate surgical intervention, such as esophagotomy with primary repair, if there is pleural or mediastinal contamination [1]. Due to their sharp, pointed morphology, fish bones are associated with a notably high risk of esophageal perforation [2,8]. Esophageal perforations caused by fish bones typically manifest as linear or circumlinear calcified lesions with adjacent areas of emphysema, fluid collection, or abscesses [9]. Although less frequently described, similar complications may plausibly occur with other ingested bones, such as beef bones. Given the potentially life-threatening consequences of misdiagnosis, distinguishing true esophageal perforations from benign anatomical mimics such as ligamentum arteriosum calcification is imperative.

In the present patient, the right aortic arch occurred in the absence of an aberrant left subclavian artery, and the left ligamentum arteriosum formed a vascular ring with the Kommerell diverticulum, thereby compressing the trachea and esophagus from behind. Although ligamentum arteriosum calcification associated with Kommerell diverticulum is rarely reported and its epidemiology remains unknown, Kanza et al. found that calcification usually occurred at the aortic end of the ligamentum arteriosum or at the Kommerell diverticulum near the origin of an aberrant left subclavian artery [10]. Given that calcifications of the ligamentum arteriosum are not uncommon, it is conceivable that they may be found incidentally on an imaging study. Since the ligamentum arteriosum in a vascular ring is located near the esophagus, its calcification can appear close to the esophageal wall. Hence, in the presence of chest symptoms and a linear pattern of calcification, the possibility of an esophageal perforation due to obstruction by a Kommerell diverticulum should be considered. An accurate diagnosis can be made by assessing the location of the ligamentum arteriosum and the absence of inflammation-related clinical findings, such as pneumomediastinum and regional fatty infiltration, on CT.

In the present case, the patient’s symptoms resolved spontaneously without intervention, and she remained clinically stable without signs of infection or complications on follow-up, further supporting the diagnosis of ligamentum arteriosum calcification rather than true esophageal perforation.

## Conclusions

Esophageal perforations caused by foreign bodies require prompt intervention, but careful interpretation of CT findings can distinguish them from ligamentum arteriosum calcification associated with a Kommerell diverticulum. Accurate imaging is essential to avoid unnecessary surgical procedures.
